# Supplementary figures and images for: Enhancing household soybean processing and utilization in the Eastern Province of Zambia, a concurrent triangulation study design
Source: PLoS One. 2023 Sep 28;18(9):e0282762. doi: 10.1371/journal.pone.0282762 (PMC10538773; doi:10.1371/journal.pone.0282762)

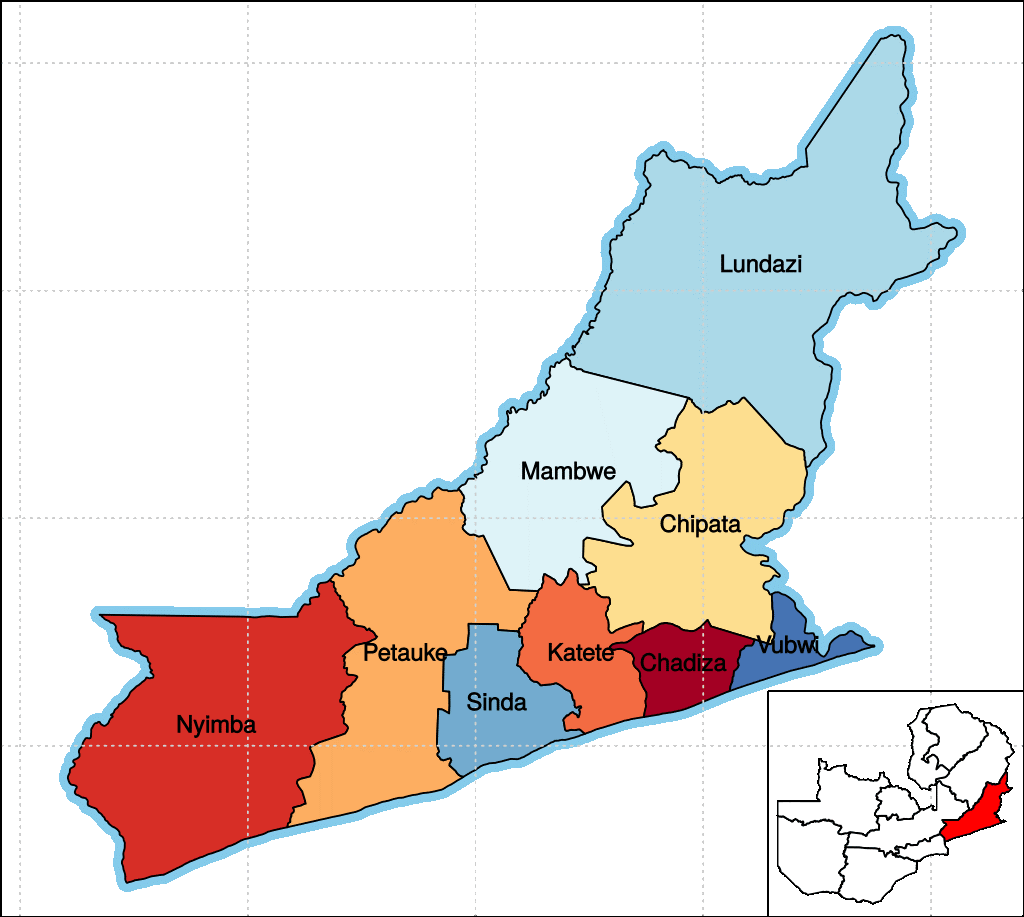

Supplement: S1 Fig — (PNG) [file pone.0282762.s001.png]
