# Supplementary material for: Enhancing household soybean processing and utilization in the Eastern Province of Zambia, a concurrent triangulation study design
Source: PLoS One. 2023 Sep 28;18(9):e0282762. doi: 10.1371/journal.pone.0282762 (PMC10538773; doi:10.1371/journal.pone.0282762)
Supplement: Attachment — Submitted filename: PLOS One Paper Review April 2023.docx [file pone.0282762.s003.docx]

The paper entitled enhancing household soybean processing in Eastern Province of Zambia, a concurrent triangulation study design though covers good understanding on subject regarding soybean household processing in Eastern Province of Zambia but it lacks defined objectives of investigative study.

The designed query has several technical flaws. For instance, though soybean is rich in well balanced essential amino acid content but it lacks sulfur containing essential amino acids methionine. It is rich in anti nutrients which is a serious matter of concern as presence of anti-nutrients interferes with the absorption of digested nutrients in the human body. Above all, anti nutrients are not completely removed by common domestic processing method. Prolong soaking of soybean seeds and soybean fermentation are two important methods which are being used to decrease the anti nutrients content of soybean seeds but this requires technical expertise which most of the household individuals lack.

In simple words even if soybean seeds yield increases several folds, the consumption of domestically processed soybean products is not adequately nourishing on nutritive grounds which would be a serious matter of concern for sustaining natural health in the Zambia s soybean domestically processed products consumers population in long run.

As far as soybean oil is concerned, maize which is another major crop of Zambia, produces more healthy oil by using technically simpler method in cost effective manner.

Method of data collection in the submitted work is not scientifically authentic and lacks the provision of identifying and limiting the errors which questions the reproducibility of the investigative work

On the basis of above mentioned grounds, I would recommend major revision of the submitted work while addressing the above raised points.
