# Supplementary material for: Enhancing household soybean processing and utilization in the Eastern Province of Zambia, a concurrent triangulation study design
Source: PLoS One. 2023 Sep 28;18(9):e0282762. doi: 10.1371/journal.pone.0282762 (PMC10538773; doi:10.1371/journal.pone.0282762)
Supplement: Attachment — Submitted filename: Review.doc [file pone.0282762.s004.doc]

**Review: Enhancing household Soybean Processing and Utilization in the Eastern Province of Zambia, a concurrent triangulation study design**

**1. Title:** The title is adequate.

**2. Abstract:** The abstract is adequately addressed.

**3. Introduction:** The introduction is adequate.

**4. Basic Reporting**

The English language structure is professional, clear and unambiguous throughout except in few lines.

The authors were consistent with one style of paragraph.

**Page 10:** The equation by Yamane (1967) is not sharp, do the needful.

All Table titles should have colon after the number before title. For example; Table 1: ……

Thank you for providing literature and references

**5. Experimental design**

The research is original primary one and within [Aims and Scope](https://peerj.com/about/aims-and-scope) of the Journal.

Research question is well defined, relevant & meaningful and it stated how research fills an identified knowledge gap. The experimental design was clearly stated. Rigorous investigations were performed to a high technical & ethical standard.

**6. Validity of the Findings**

Impact and novelty are assessed. Negative/inconclusive results accepted. Meaningful replication was encouraged since the experimental design was clearly stated; rationale & benefit to literature is clearly stated.

All underlying data have been provided; they are robust, statistically sound, & controlled.

**Conclusion:** This should address the summary of the objectives outlined for the study, linked to original research question & limited to supporting results. Conclusions are not well stated,

Thus the authors should recast this.
