# Supplementary material for: Enhancing household soybean processing and utilization in the Eastern Province of Zambia, a concurrent triangulation study design
Source: PLoS One. 2023 Sep 28;18(9):e0282762. doi: 10.1371/journal.pone.0282762 (PMC10538773; doi:10.1371/journal.pone.0282762)
Supplement: Attachment — Submitted filename: Review 2023 April.docx [file pone.0282762.s006.docx]

The revised paper entitled Enhancing household Soybean Processing and Utilization in the Eastern Province of Zambia, a concurrent triangulation study design fails to accommodate all aspects of academic concerns raised in my earlier review.

The manuscript lacks scientifically designed objectives of the study besides indicating inadequate literature review on relevant aspects of the topic while over emphasizing on statistical analysis of inappropriate and insufficient data which can mislead the drawn conclusion.

A very important aspect which is found missing in the investigative study was to evaluate the effect of different domestic processing methods on Soybean s Anti Nutrient Contents and on Nutritional Composition of different Domestically Produced Soybean Products. It would be helpful if Average Diet Intake Menu Compositions ( also in terms of nutritional composition) of an adult ( Man and Woman respectively) and a child ( Boy and Girl respectively ) are mentioned in the paper, a pre requisite to assess the suitability of the Domestically Prepared Soybean Products, discussed in the paper, for adding in Daily Diet Intake Menu of adults and children while avoiding the risk of mal nutrition in them in long run. There is an utmost need to add and to discuss in the paper the efficiency and reliability of methods being recommended for domestic use to remove Anti-Nutrients from Soybean Products referred in the paper after assaying their effects on nutritional composition and digestibility of the Soybean Products to be prepared domestically.

It is important to discuss in the paper why have Soybean based Domestically Prepared Products been preferred for study to assess their suitability for regular use over other comparable agricultural products which can be grown cost effectively and are suitable for domestic processing but lacks Anti Nutrients.

On the basis of my above mentioned findings, I would recommend major revision of manuscript while addressing the points mentioned in this review of the Submitted Revised Manuscript.
