# Supplementary material for: Enhancing household soybean processing and utilization in the Eastern Province of Zambia, a concurrent triangulation study design
Source: PLoS One. 2023 Sep 28;18(9):e0282762. doi: 10.1371/journal.pone.0282762 (PMC10538773; doi:10.1371/journal.pone.0282762)
Supplement: Attachment — Submitted filename: Response to Reviewers.docx [file pone.0282762.s008.docx]

Levy Mwanawasa Medical University

Box 33991

Lusaka

Zambia

8^th^ August 2023

The Editor

PlosOne

Dear Sir/Madam,

**SUB: REBUTTAL LETTER - RESPONSE TO REVIEWERS**

The subject matter above refers.

This serves to outline the responses and action taken for the points raised by the academic editor and the reviewers with details in the table below for your consideration.

| Point raised by the academic editor and reviewer(s) | Action |
| --- | --- |
| Please rewrite the study's objective in full sentences rather than as a subheading. This is not a dissertation. | This has been done. It is highlighted in yellow at the end of the introduction, just before methodology in the “Revised Manuscript with Track Changes”. |
| In addition, because you have not yet responded to the comment on editing, the paper requires extensive language editing. | Extensive language editing has been done throughout the paper. |

Looking forward to having the paper published.

Yours faithfully,

Priscilla Funduluka

**Author**
