# Supplementary material for: Enhancing household soybean processing and utilization in the Eastern Province of Zambia, a concurrent triangulation study design
Source: PLoS One. 2023 Sep 28;18(9):e0282762. doi: 10.1371/journal.pone.0282762 (PMC10538773; doi:10.1371/journal.pone.0282762)
Supplement: Attachment — Submitted filename: Reviewer Comments.doc [file pone.0282762.s005.doc]

Reviewer Comments:


The authors revised the manuscript and incorporated all the corrections in the revised manuscript.
